# Supplementary material for: IFN-γ signaling is required for the efficient replication of murine hepatitis virus (MHV) strain JHM in the brains of infected mice
Source: PLoS One. 2025 Jun 5;20(6):e0317482. doi: 10.1371/journal.pone.0317482 (PMC12140286; doi:10.1371/journal.pone.0317482)
Supplement: S2 Table — (PDF) [file pone.0317482.s006.pdf]

Table S2. qPCR primers

	Forward 5'→3'	Reverse 5'→3'
HPRT	GCGTCGTGATTAGCGATGATG	CTCGAGCAAGTCTTTCAGTCC
IFN-β	TCAGAATGAGTGGTGGTTGC	GACCTTTCAAATGCAGTAGATTCA
IFN-γ	CGGCACAGTCATTGAAAGCCTA	GTTGCTGATGGCCTGATTGTC
CXCL-10	GCCGTCATTTTCTGCCTCAT	GCTTCCCTATGGCCCTCATT
IL-1β	ACTGTTTCTAATGCCTTCCC	ATGGTTTCTTGTGACCCTGA
IL-6	GCTACCAAACTGGATATAATCAGGA	CCAGGTAGCTATGGTACTCCAGAA
PARP1	CAGGAGAGTCAGCGATCTTGG	ACCCATTCCTTTCGGCTAGG
PARP2	TGGAAGGCGAGTGCTAAATG	GGGCTTTGCCCTTTAACAGC
PARP3	TGCGGCATGTTTGGAAAGTG	GTGCATGGTGGTAACATAGCC
PARP4	AGTGCTACAGCCCGTTTCC	CACAGCTTTCAGTTGTGGGC
PARP5a	CCCTGAGGCCTTACCTACCT	TCAAGACCCGCAACTTCTCC
PARP5b	TGATGGCAGAAAGTCAACTCCA	GCCACAGGTCCATTGCATTC
PARP6	GTACCTTGATGGACCAGAGCC	GCCAGCTCGGAACTTCTTGA
PARP7	ATTTACAGACACTTGGTGGGG	GGCACTTGGATGAAGTCCTGA
PARP8	CACTTCCGAAACCACTTCGC	TAGGATACACTTTTGGGGCCG
PARP9	GCATTTGCTAAAGAGCACAAGGA	AAAGCACCACTATTACCGCTGA
PARP10	CGAAACGGCACACTCTACGG	GAGACCCTCAAAGGAGGTGC
PARP11	GGCTGTCTTTGGAAAAGGAACC	GCACTCGAGCAAGAAACATGG
PARP12	AGACCGGGAAGAACTGTAGGA	TTTGGAAGGAGCAAGAGCCG
PARP13	AGTAGTCCCACTGGTTTTGGC	TGCAACTCTGTGGCTTGTGG
PARP14	TGCTGAAGCTGTCAAGACTACA	ACAATGGCATGGGTCGTAGC
PARP16	CTTTGACCCGGCCAACTCC	AAACAGAGAAGTCTTGTTCAGGTG
genomic RNA	AGGGAGTTTGACCTTGTTCAG	ATAATGCACCTGTCATCCTCG
